# Understanding and overcoming barriers to digital health adoption: a patient and public involvement study

**DOI:** 10.1093/tbm/ibaf010

**Published:** 2025-04-01

**Authors:** Jacqueline Louise Mair, Jumana Hashim, Linh Thai, E Shyong Tai, Jillian C Ryan, Tobias Kowatsch, Falk Müller-Riemenschneider, Sarah Martine Edney

**Affiliations:** Future Health Technologies, Singapore-ETH Centre, Campus for Research Excellence And Technological Enterprise (CREATE), 1 Create Way, Singapore, 138602, Singapore; Behavioural and Implementation Science Interventions, Yong Loo Lin School of Medicine, National University of Singapore, 10 Medical Drive, Singapore, 117597, Singapore; Centre for Digital Health Interventions, Department of Management, Technology, and Economics, ETH Zurich, Weinbergstrasse 56/58, 8006, Zurich, Switzerland; Behavioural and Implementation Science Interventions, Yong Loo Lin School of Medicine, National University of Singapore, 10 Medical Drive, Singapore, 117597, Singapore; Saw Swee Hock School of Public Health, National University of Singapore, 12 Science Drive 2, Singapore, 117549, Singapore; Future Health Technologies, Singapore-ETH Centre, Campus for Research Excellence And Technological Enterprise (CREATE), 1 Create Way, Singapore, 138602, Singapore; Saw Swee Hock School of Public Health, National University of Singapore, 12 Science Drive 2, Singapore, 117549, Singapore; Behavioural and Implementation Science Interventions, Yong Loo Lin School of Medicine, National University of Singapore, 10 Medical Drive, Singapore, 117597, Singapore; Painted Dog Research, 658 Newcastle Street, Leederville, WA 6007, Australia; Institute for Implementation Science in Health Care, University of Zurich, Universitätstrasse 84, 8006, Zurich, Switzerland; School of Medicine, University of St. Gallen, St. Jakob-Strasse 21, 9000, St. Gallen, Switzerland; Centre for Digital Health Interventions, Department of Management, Technology, and Economics, ETH Zurich, Weinbergstrasse 56/58, 8006, Zurich, Switzerland; Future Health Technologies, Singapore-ETH Centre, Campus for Research Excellence And Technological Enterprise (CREATE), 1 Create Way, Singapore, 138602, Singapore; Saw Swee Hock School of Public Health, National University of Singapore, 12 Science Drive 2, Singapore, 117549, Singapore; Digital Health Center, Berlin Institute of Health, Charité-Universitätsmedizin Berlin, Charitépl. 1, 10117, Berlin, Germany; Saw Swee Hock School of Public Health, National University of Singapore, 12 Science Drive 2, Singapore, 117549, Singapore

**Keywords:** public health, minority groups, digital divide, health promotion, health behavior

## Abstract

**Background:**

Digital health (DH) technologies provide scalable and cost-effective solutions to improve population health but face challenges of uneven adoption and high attrition, particularly among vulnerable and minority groups.

**Purpose:**

This study explores factors influencing DH adoption in a multicultural population and identifies strategies to improve equitable access.

**Methods:**

Using a Patient and Public Involvement approach, lay facilitators engaged adults at public eateries in Singapore to discuss motivations and barriers to DH adoption. A semi-structured guide facilitated discussions, followed by an optional socio-demographic survey. Data were analyzed through inductive thematic analysis and mapped to behavior change theory to identify mechanisms of action (MoA) and behavior change techniques (BCTs) to support adoption.

**Results:**

Facilitators engaged 118 participants between November 2022 and February 2023. Five key themes were identified from the discussions: (a) awareness of DH solutions, (b) weighing benefits against burdens, (c) accessibility, (d) trust in DH developers and technology, and (e) the impact of user experience. These themes were mapped to 13 MoA and 26 BCTs, informing five key strategies to enhance DH adoption: community-based promotion of credible DH solutions and digital literacy training, brief counselling at opportune moments in healthcare settings, variable rewards tied to personal values, policies ensuring accessibility and regulation, and gamified, user-friendly designs emphasizing feedback and behavioral cues.

**Conclusion:**

Designing and implementing DH solutions that are accessible, trustworthy, and motivating—integrated within healthcare services and promoted through community efforts—can address barriers to adoption by diverse communities and may help to narrow the digital divide.

## Teaser

Despite the promise of digital health solutions, adoption remains uneven, particularly among vulnerable communities. This study identifies key barriers and strategies to improve equitable access and engagement in multicultural populations.

**Implications Statements**

Practice: The design and wide-scale implementation of accessible, motivating, trustworthy, and user-friendly digital health solutions, that are integrated within healthcare and community settings, will address barriers to adoption by diverse communities and may help narrow the digital divide.

Policy: Policymakers who want to increase the uptake and sustained use of digital health on a population level should consider community-based support and promotional activity, integration of brief counselling and relevant resources within healthcare settings, the provision of variable and sustainable reward structures, establishing regulatory approval processes to build trust, and prioritizing user experience during development phases.

Research: Future research should be aimed at (a) developing and implementing scalable and accessible digital health solutions that can adapt to the diverse needs and preferences of individuals and (2) better understanding the relationships between digital health engagement and health behavior change, and how these vary over time.

## Introduction

The development and proliferation of digital health (DH) technologies have shown promise in revolutionizing the way people access health information, adopt healthier behaviors and manage their health. From telemedicine and wearable devices to mobile apps and AI-driven tools, these innovations have the potential to support widespread health promotion and improve population health outcomes at scale [1]. Indeed, DH solutions that target lifestyle behaviors for the prevention or management of noncommunicable diseases are plentiful. Yet, their adoption remains unevenly distributed, often leaving behind those who are disadvantaged by health, economic, cultural, or social conditions but who stand to benefit the most [2, 3]. For example, factors such as limited internet access, lower technology confidence, language barriers, and mistrust contribute to their under-representation in DH adoption [4]. Even among those who adopt, for example, health apps, sustaining long-term engagement and adherence remains a critical challenge [5, 6]. Research indicates that while initial uptake may be high, usage often declines sharply, possibly due to loss of motivation, lack of personalization, and poor user experience [7–9]. Addressing these challenges is essential for maximizing the long-term impact of DH solutions and ensuring that they reach those who need them.

Current evidence on DH adoption is largely from high-income Western countries where social, cultural, and environmental influences on health behaviors differ significantly from other regions, such as Asia [5]. The evidence on user preference and satisfaction with DH solutions also comes from small-scale or controlled trials with narrow eligibility criteria rather than large-scale public health programs that serve more diverse populations, making it difficult to generalize findings or assess acceptability in real-world contexts. Moreover, DH research often underrepresents socioeconomically disadvantaged groups, including racial and ethnic minorities, and other vulnerable populations [10, 11] limiting our insights to the “worried well” and exacerbating the digital divide [12, 13].

Singapore offers a unique setting to explore DH adoption. Firstly, smartphone penetration is among the highest globally at 97% [14], therefore most of the population has lived experiences using mobile technology. Additionally, as part of its “Smart Nation” initiative, Singapore has integrated technology into nearly every aspect of life—business, health, transport, urban living, and government services—exposing its residents to high levels of technology engagement [15]. Secondly, preventive health is a priority for the government [16], giving residents access to numerous health promotion programs, both online and offline, often free or at low cost [17]. Finally, Singapore’s diverse, multicultural population provides insights into how social and cultural factors, including language, dietary habits, and religious beliefs, shape daily lifestyle choices and health behaviors.

This study aims to explore the factors influencing DH adoption in a diverse and multicultural population and propose strategies to improve the accessibility and adoption of DH across Southeast Asia. Our work is grounded in the belief that DH, with its ability to transcend geographical and temporal barriers, should play a central role in the future of health promotion for all.

## Methodology

A Patient and Public Involvement (PPI) narrative approach [18, 19] was used to engage the local community and capture the voices of people from a broad range of backgrounds. This approach reduces barriers to research participation, ensuring valuable insights from all members of the public are heard. The study was conducted between November 2022 and February 2023 and was approved by the Institutional Review Board of the National University of Singapore (NUS-IRB-2021-956). The study conforms to standards for reporting qualitative research (Supplementary file 1).

### Setting

The study was conducted in Singapore, a multi-ethnic society of 5.64 million where most residents live in public housing [20]. Hawker centres, public dining spaces offering affordable food, are key community hubs. These hawker centres provide an ideal setting to engage residents in a casual environment, facilitating open and informative dialogue with those less likely to participate in research.

### Study team

The research team was made up of (1) a core team including two co-principal investigators (experienced in mixed-methods digital health behavior research), a PhD researcher (experienced in co-production and PPI research methods), and a research assistant (Bachelor of Science), and (2) an advisory team including three professors with expertise in digital health research and one research collaborator experienced in PPI research.

Six lay facilitators were recruited through a university internship program to interact with members of the public at hawker centres under the research team’s supervision. Facilitators were all female university students, who spoke a variety of local languages (English, Malay, Tamil, and Chinese, including Mandarin, Hokkien, Teochew, and Cantonese) and received hourly wages for their participation. To ensure consistency and adherence to study protocols, they underwent training on study procedures and interview techniques. They were also actively involved in developing the interview topic guide and data capture template.

All had an interest in DH, but none had any relationship with the participants in the study.

### Participants

The target sample included Singapore residents aged 21 years or older. Nonrandom sampling was used to facilitate the inclusion of diverse perspectives. Locations were chosen based on the proportion of residents with lower socioeconomic status, the number of dining outlets catering to ethnicity-linked dietary requirements (such as Halal), and proximity to places of worship frequented by ethnic minorities. Inclusivity was favoured over proportional population representation based on age, gender, and ethnicity. Therefore, lay facilitators were instructed to over-sample certain groups (e.g. Malay and older adults) when necessary. Visits occurred at different timings in the morning-noon (9:00AM–12:00PM) and late afternoon-evening (5:00PM–8:00PM) periods on weekdays and weekends to include both working and nonworking participants.

### Study procedure

Insights from relevant health behavioral change models [21], similar exploratory studies on health behaviors and disease prevention [22, 23], and lay facilitator perspectives were used to construct an interview topic guide (Supplementary File 2) and corresponding data capture template. Questions covered participants’ health-related needs and routines, familiarity with existing DH programs and apps, motivations and barriers to DH adoption, and suggestions to improve uptake and sustained use.

Following the PPI Hawker method outlined by Luna Puerta and colleagues [18], pairs of lay facilitators approached people at hawker centres to discuss DH, ensuring only individuals who were not eating were approached. The pairing of facilitators was flexible based on their availability for each session. Following a brief study overview and obtaining verbal consent, participants were asked about their familiarity with a range of popular DH programs and apps in Singapore (Supplementary File 3). The conversation flow then varied based on the participant’s experience, typically falling into: (i) current DH user, (ii) previous DH user, (iii) aware of DH but never used, or (iv) unaware of DH solutions. One facilitator led the discussion using a semi-structured interview guide, while the other took notes. Conversations lasted between 5 and 10 minutes, followed by an optional anonymous sociodemographic questionnaire available in English and Mandarin (Supplementary File 3). For participants who declined to complete the questionnaire, facilitators estimated their age group, sex (male or female), and ethnicity (Chinese, Malay, Indian, or other). As a token of appreciation, participants were offered a free beverage for their time.

Following each session, facilitators reviewed their notes, reflected on their interactions, and added any necessary details. Their notes were discussed with the research team, along with any feedback on the process. This collaborative review allowed the team to reach consensus on the depth and breadth of the discussions, refine questions for future sessions, and ensure data quality. Data saturation was determined using three criteria: (i) a comprehensive understanding of emerging topics, (ii) a sufficiently diverse range of perspectives, and (iii) three consecutive sessions in which no new topics were identified [22]. Additional prompts were iteratively introduced to ensure thorough coverage of DH, particularly regarding diet management and mental well-being apps, and to elicit more detailed insights into participants’ needs and decision-making when engaging with DH programs and apps.

### Data analysis

All discussion content was recorded in a standardized field note template and linked to the corresponding demographic questionnaire using a unique ID. Notes were organized under the four predetermined categories of DH experience, with some participants contributing to more than one category (e.g. discussing a health app they currently use while also reflecting on one they had stopped using).

Sociodemographic data were coded and summarized using descriptive statistics. Facilitator field notes were digitized and analysed using qualitative analysis software (ATLAS.ti, version 24.0.1). We employed a hybrid approach to analysis whereby relevant themes were identified through inductive thematic analysis [24], and then deductively mapped to behavioral change theory using the Theory and Techniques Tool [25]. The analysis was conducted from an essentialist/realist viewpoint [26], aiming to provide clear and straightforward descriptions of the phenomenon of interest, using language closely aligned with the collected data. Firstly, field notes were read several times before codes were generated by one researcher (JLM) and sorted into potential themes in collaboration with a second researcher (JH). Initial themes and results were presented to lay facilitators during a debrief to validate the findings and identify any misinterpretation or missing information. Codes and themes were further refined and mapped to MoA using the Theory and Techniques Tool to identify potential behavioral change techniques (BCTs) that could support DH adoption. One researcher (JLM) conducted the initial mapping which was reviewed by a second researcher (JH) to ensure accuracy and consistency. Only BCTs with empirical evidence linking them to the identified MoA were included, and their relevance was cross-checked against the themes derived from the data. Based on this mapping, one researcher (JLM) formulated potential strategies for implementing these BCTs in a way that could activate the corresponding MoA and address the identified themes. As patterns emerged, these strategies were grouped and refined to develop five key recommendations for enhancing DH adoption. For narrative flow, quotes from the facilitators’ notes are presented in the participants’ voices.

## Results

Lay facilitators approached 240 individual participants, with 118 (49%) agreeing to take part in a discussion. Five participants were later excluded from the analysis for not meeting the eligibility criteria (*n* = 2 tourists, *n* = 2 underage) or due to loss of field notes (*n* = 1). The recruited sample (46% male; 43% aged 21–40, 30% aged 41–60, 27% aged >61 years; and 64% Chinese, 14% Malay, 16% Indian and 6% other ethnicity), largely represented the Singaporean population compared to Census 2022 data (Table 1). There was an even distribution of people with experience using DH technologies (*N* = 76; *n* = 53 current users, *n* = 23 previous users) and those without experience (*N* = 80; *n* = 51 aware of DH but do not use, *n* = 29 not aware of DH solutions) [some participants’ fell into more than one category due to their experience with different DH solutions].

**Table 1:** Sample characteristics.

		All (*n* =113) (%)^*^	Census 2022 (%)	DH users (%)		Non DH users (%)	
				Current (*n* = 53)^*^	Previous (*n* = 23)^*^	Aware (*n* = 51)^*^	Not Aware (*n* = 29)^*^
Gender (*n* = 113)†	Male	46	49	42	53	49	48
	Female	54	51	59	48	51	52
Age group (years) (*n* = 113)†	21-40	43	28	47	57	41	48
	41-60	30	30	28	17	35	24
	>60	27	24	25	26	24	28
Ethnicity (*n* = 113)†	Chinese	64	74	66	70	59	62
	Malay	14	14	15	13	20	10
	Indian	16	9	15	9	14	21
	Other	6	3	4	9	8	7
Education (*n* = 105)	Degree	34	36	45	48	29	24
	A Level/ Diploma	37	27	38	30	41	35
	Secondary	16	16	11	17	16	24
	Primary	6	21	2	0	6	10
Monthly Household Income SGD (*n* = 82)	<4000	36	36	31	26	40	38
	4000–5999	18	10	25	22	16	17
	6000–9999	12	18	15	9	8	10
	>10 000	6	48	6	13	4	0
Housing (*n* = 105)	≤3 room HDB	21	24	15	13	24	38
	4 or 5 room HDB	51	55	58	48	48	38
	Private condominium	13	17	15	30	12	3
	Private landed house	4	5	6	0	2	7
	Other	5	NA	2	0	8	10

^*^Values may not add to 100% due to rounding or missing data; sample sizes per category may not tally due to participants’ views/experiences falling into more than one category; study age exclusion <21 years. †*n* = 4 gender, age group, and ethnicity were estimated by the facilitator. DH, digital health, SGD, Singapore Dollars (1 SGD = approximately 0.74 USD), HBD, housing development board (government-subsidised housing, >80% of Singapore’s population live in HBD apartments).

### Themes

Participants discussed both positive and negative perceptions and experiences regarding the use of DH, which are described within five themes: (a) awareness of what DH solutions offer, (b) weighing the benefits against the burdens, (c) accessibility, (d) trust in the entity behind the DH solution and its capabilities, and (e) the impact of user experience.

#### Awareness of what DH solutions offer

Lack of awareness of available DH programs and apps was one of the most common reasons for not using them. Participants suggested that people are “not aware of the programs because people are not exposed enough” (P77, Male, Chinese, aged 31–40 years) or they “don’t know the benefits of the app, so more promotion can be done to promote apps and their benefits” (P71, Female, Chinese, aged 41–50 years). Despite many praising the government for their efforts to promote healthy living and offer DH programs, some felt the “government needs to have more campaigns to raise awareness” (P130, Male, Chinese, aged 31-40 years). Strategies to increase awareness were suggested, including promotion in the community, such as in shopping malls, transport stations, community centres and church groups, as well as more traditional radio or television advertising because an “old-fashioned way of advertising can give a lot of exposure to everyone (especially older generations)” and “apps need to go viral [on social media] before people will notice them” (P89, Female, Chinese, aged 51-60 years). One of the most common reasons for DH uptake was referral from a trusted person, such as a family member, friend, employer, or health professional. This could be in the form of a recommendation to use an app (“my wife encouraged me to use the [Healthy365] app” P36, Male, Indian, aged 61-70 years), the gifting of a wearable device (“I got an old version of the Fitbit tracker from a friend and noticed it was good, so I invested in a new one” P61, Female, Malay, aged 41–50 years), encouragement to participate in a program or challenge with others (“family member heard [of National Steps Challenge] from a friend and wanted the entire family to try together” P80 Male, Chinese, aged 21–30 years), or based on health advice from a doctor (“told by my doctor to lose weight and exercise more” P35 Female, Chinese, aged 61–70 years).

#### Weighing of benefits and burdens

A key driver of DH uptake and sustained use was the perception that “people will only be inclined to use an app if they are aware that it benefits them somehow” (P74, Female, Malay, aged 21–30 years). Apparent in the data was the phenomenon of a constant “weighing” of perceived needs, benefits, and burdens and whether one sees overall value in using a DH program or app. In other words, “do I want this?,” “does it fulfil my needs?,” “what do I get out of it?,” and “is it worth the effort?.”

Firstly, a key consideration in the uptake of a DH solution is whether individuals perceive their basic need for “good health and wellbeing” as already fulfilled. For example, participants who perceived themselves to be leading a healthy lifestyle did not see the need for a health app; “I don’t track calories as I know what I’m eating and I don’t need it” (P68, Female, Chinese, aged 31-40 years), “I exercise every day at home and do not feel the need to use any applications to track my exercise” (P3, Male, Chinese, aged 51-60 years), “I don’t need any mental health apps since I don’t have issues regarding mental health” (P115, Male, Chinese, aged 31-40 years). For others, using a DH solution is borne out of a desire to monitor and receive feedback on their health behaviors, such as step counts, exercise, calorie intake, sleep, or heart rate, as well as progress towards predefined goals over time. This could be motivated by a concern for one’s health status (e.g. “my device tracks sleeping patterns to monitor how I sleep because I have irregular heartbeat. If there is anything wrong, I can check immediately” P69, Female, Chinese, aged 31-40 years) or a perceived need for support in maintaining healthy behavior (e.g. “it provides a visualization of daily workouts to motivate me” (P8, Male, Chinese, aged 61-70 years). While such information can be helpful to some, it can have the opposite effect on others: “it is unhealthy…I became obsessed about counting calories” (P56, Female, Other, aged 21–30 years) and “health apps can make users paranoid as they are worried they will not achieve the goal” (P90, Female, Malay, aged 41–50 years). The desire to use DH could also be dynamic, in the sense that the technology may serve a purpose for some time, but once behavior change has occurred or health status improved, it may no longer be needed; “once I knew how to do meditation, I didn’t need the app anymore” (P118, Female, Chinese, aged 61–70 years).

Another important driver of uptake is whether the perceived benefits of a DH solution outweigh the perceived costs of using it. Benefits could be intrinsic, such as health monitoring and receiving feedback (as described above), or extrinsic in the form of incentives (e.g. free activity trackers) and rewards (e.g. exchanging points for shopping or transport vouchers), while costs were largely centred around time burden, financial considerations, or the effort required to engage with an app.

The initial process of downloading and registering to use a health app, including getting access to activity trackers (even when offered for free), was often perceived as “too tedious” (P89 Female, Chinese, aged 51–60 years), and some were unmotivated to even start the process: “I’m lazy to go and get the free activity tracker as the process takes time” (P130, Male, Chinese, aged 31–40 years). For many, the technology itself is a barrier. Participants cited a perceived “lack of time to use health apps” (P13, Male, Indian, aged 31–40 years) or that they “refrain from using technology” because they “assume a lot of effort is needed to use the apps” (P16, Female, Indian, aged 61–70 years). Even for those currently using DH, it was noted that “[apps] can be helpful if used, but it is troublesome to keep using them, and you need to find time to do it and open the app” (P92, Male, Malay, aged 21–30 years). In such examples, the perceived costs of use outweigh the benefits, therefore people do not adopt them.

For others, however, the perceived burdens are worth the effort. For example, many people “track steps to collect points and get vouchers” (P41, Female, Chinese, aged 51–60 years) with some highlighting this as a “competitive edge” over other health apps (P48, Male, Chinese, aged 51–60 years) and that they “would not use the apps if no rewards are given” (P132, Male, Chinese, aged 21–30 years). However, monetary incentives need to be “attractive enough” (P89, Female, Chinese, aged 51–60 years) to different people to motivate health app use, and if it “takes too long to accumulate enough points for redemption” (P80, Male, Chinese, aged 21–30 years) or incentives are “insufficient” (P26, Female, Chinese, aged 51–60 years) it can lead to abandonment of the app. Finally, for sustained use, appraisal shifts towards whether the expected benefit is realized and is worth the actual effort involved. For example, one participant explained, “I started to use [app] because I know the good effect about meditation, but found it hard to see benefits, so I quit” (P114, Male, Chinese, aged 21–30 years).

#### Accessibility

Poor digital literacy was frequently identified as a barrier to the adoption of DH. For instance, one participant stated she “does not know how to use a [smart]phone” (P126, Female, Malay, aged 61–70 years), while another described herself as “not app-savvy” (P58, Female, Indian, aged 51–60 years). Another participant expressed disinterest in learning how to use apps, stating, “I’m afraid I will forget” (P27, Female, Chinese, aged 71–80 years). It was suggested that “health apps should be made to cater to any age group – making it easier to use, especially for the really old and young age groups” (P76, Female, Malay, aged 41–50 years). Additionally, the language barrier was noted as a significant issue, as many apps are primarily available in English. Participants suggested that offering apps in “multiple languages, such as Mandarin” (P19, Male, Chinese, aged 21–30 years), and providing multilingual informational leaflets for older users would help improve accessibility and increase adoption.

The digital divide is further evidenced by the upfront and ongoing costs associated with using DH solutions. Certain apps are only accessible to users who own specific devices, such as an Apple Watch, or smartphones with sufficient storage capacity, or who have data plans robust enough to use an app without relying on WiFi. For example, health apps that require syncing with a specific smartwatch or wearable tracker exclude those who “cannot afford an Apple Watch” (P73, Male, Malay, aged 21–30 years). Even without the need for connected wearables, some people would “hesitate if [a health app] costs money” (P111, Male, Filipino, aged 41–50 years) and are reluctant to make “unnecessary purchases in the name of wellbeing” (P70, Female, Indian, aged 61–70 years). Several individuals highlighted that their decision to participate in Singapore’s National Steps Challenge was based on the free wearable activity tracker that was offered. However, the quality and durability of this free device influences their continued participation because participants are unwilling to “spend money if the watch spoils” (P87, Male, Malay, aged 41–50 years) to repair or replace it. Moreover, continued use of an app is often hindered by ongoing or additional costs, particularly in freemium models where basic features are free, but premium features require payment. One participant mentioned discontinuing the use of an app because “the features I wanted to use required a subscription” (P122, Female, Chinese, aged 21–30 years).

#### Trust

Trust in the individual recommending a DH solution and in the technology itself (whether it looks like a legitimate product or not) influences whether one feels it is safe to download a health app and enrol in a DH program. Some participants are “very cautious and sceptical about apps, and won’t anyhow download apps” (P105, Male, Chinese, aged 61–70 years) and find that “some apps can be fake” and “should have more security” (P64, Female, Chinese, aged 31–40 years). However, if one trusts the person or organization promoting an app, then the likelihood of uptake is higher. For example, many participants started using a DH solution because they were “recommended by many friends” (P43, Male, Chinese, aged 51–60 years) or their “family introduced the app” (P33, Female, Chinese, aged 61–70 years [estimated]). Uptake is also enhanced if referred by an authoritative figure such as a health professional or even the government; one participant stated “I follow my doctor’s orders strictly. If the doctor recommends an app to me, I will use it” (P28, Female, Malay, aged 51–60 years), while another “will consider trying digital health apps if they are being promoted by the government” (P117, Female, Chinese, aged 31–40 years). Additionally, trust in the accuracy of the health tracking data provided by a DH solution appears to be crucial for continued use. For example, discrepancies in step count between different apps, inaccurate distance tracking, and glitches with data syncing properly, were among some of the reasons for app abandonment.

#### User experience

Sustained use of a DH solution is largely influenced by a continuous appraisal of the perceived benefits versus the associated burdens or costs. This appraisal process is ongoing, and key factors shaping it over time include issues related to compatibility, usability, and performance. Many users noted that not owning a wearable tracker or not having the correct type of tracker limited their ability to use certain apps. In addition, some participants found the process of syncing apps with trackers overly complex. As one user remarked, “the design and interface for pairing devices should be more user-friendly and compatible with a wider range of devices” (P32, Male, Chinese, aged 21–30 years). Usability concerns were primarily centred on poor functionality and the need for more intuitive, user-friendly interfaces. Performance problems also contributed to dissatisfaction, with participants complaining about “laggy” apps and “frustrating” auto-logouts (P80, Male, Chinese, aged 21–30 years). These technical problems were significant barriers to sustained use.

In addition to these factors, gamified user experiences emerged as a key element that could facilitate sustained DH use. One participant emphasized the appeal of “daily challenges that are different every day and relate to various behaviors” (P121, Female, Chinese, aged 21–30 years). The concept of maintaining a streak was also highlighted as crucial for keeping users engaged, with some describing it as essential for “keeping momentum” (P61, Female, Malay, aged 41-50 years).

### Mechanisms of action

Mapping the resultant themes using the Theory and Techniques tool identified 13 MoA and 26 corresponding BCTs that could improve the adoption of DH solutions (Table 2).

**Table 2:** Strategies to improve adoption of digital health, mapped to mechanisms of action with evidential links to behavior change techniques

Theme	Mechanism of action	Behavior change technique	Example strategies	Type
**Uptake**				
Awareness	Knowledge	5.1. Information about health consequences	Implement a regular series of community-based promotional events (talks, workshops, leaflets, posters) and mass media campaigns on the availability and benefits of using DH programs and apps.	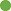
	Social influences	3.1. Social support (unspecified)	Offer a “refer a friend” incentive to increase promotion of DH programs and apps by current users.	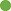
	Behavioral cuing	7.1. Prompts/cues	Promote available DH programs and apps through reminders or push notifications from other public service or health-related apps. Increase DH promotion and point-of-choice cues in places frequented by people and where smartphone use is high (such as on public transport and local food centres).	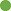
Weighing of benefits and burdens	Beliefs about consequences	5.2. Salience of consequences	Communicate the benefits of using DH solutions via influential role models or authority figures in an immersive and interactive way, such as theatre, radio, podcasts, or community talks.	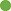
		10.1. Material incentive (behavior)	Ensure alternative reward options that appeal to different people, such as reinvestment in public facilities or charity donation, and non-financial rewards such as tree planting, are available.	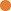
		5.1. Information about health consequences	Include a structured brief behavior counselling session combined with an informational leaflet during routine health screening for those not using DH. The content of the session should involve a discussion about the patient’s health status and risk profile and link to the health and emotional consequences of using DH, the possible pros and cons involved, expectations of DH and what changes they could support, what change would look like, and how others have successfully used DH programs and apps. Sessions should encourage users to define personal reasons for using a DH solution, goals and intentions for use, and reasonable expectations for outcomes. The information should be personalised where possible. Offer [subsidised] health monitoring devices as part of routine health services to encourage health monitoring and raise awareness of health behaviors. Combine the provision of a health monitoring devices as part of routine health screening services with brief counselling session on the benefits of health monitoring and how to interpret the feedback provided by such devices.	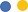
		5.5. Anticipated regret		
		5.6. Information about emotional consequences		
		9.2. Pros and cons		
		9.3. Comparative imagining of future outcomes		
	Knowledge	2.6. Biofeedback		
		5.1. Information about health consequences		
	Motivation	1.3 Goal setting (outcome)		
		9.2. Pros and cons		
		10.8. Incentive (outcome)	Promote a range of different reward option that align with different values.	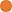
Trust	Attitude towards the behavior	9.1. Credible source	Promote, refer, or recommend DH programs and apps via trusted organizations or authority figures (such as insurers, government bodies, health professionals, or well-known community figures). Introduce a vetting system whereby only DH solutions that are approved and legally compliant (by relevant authorities and standards) can be made available to consumers and communicate the policy widely.	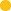
Accessibility	Beliefs about capabilities	4.1Instruction on how to perform behavior	Provide community-based digital literacy training programs.	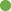
	Skills	8.1. Behavioral practice/rehearsal		
	Environmental context and resources	3.2. Social support (practical)	Offer means tested subsidies or reimbursement for DH programs and apps.	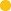
**Engagement**				
Awareness	Behavioral cueing	1.4. Action planning	Ensure DH programs and apps are designed to support self-regulation of behavior, by including health monitoring components that allow users to set health-related goals, track behaviors and outcomes and progress towards goals, highlight discrepancies between behavior and goals, support users to make detailed plans to execute healthy behaviors, prompt repetitive use of the DH solution and execution of behaviors, and provide feedback on behaviors and health outcomes. Link health monitoring data from DH solutions to patient electronic medical records so that health professionals can offer professional feedback and insights on the health data and encourage continued DH use over time.	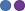
		7.1. Prompts/cues		
		8.3. Habit formation		
Weighing of benefits and burdens	Knowledge	2.6. Biofeedback		
		5.1. Information about health consequences		
	Feedback processes	1.6. Discrepancy between current behavior and goal		
		2.2. Feedback on behavior		
		2.3. Self-monitoring of behavior		
		2.7. Feedback on outcome(s) of behavior		
	Motivation	1.3 Goal setting (outcome)		
		10.10. Reward (outcome)	Provide users with tailored rewards that align with their core values.	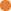
	Reinforcement	10.2. Material reward (behavior)	Reduce barriers to reimbursement of rewards by, for example, aligning time frames with achievable personalized goals.	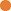
Trust	General attitudes/beliefs	9.1. Credible source	DH solutions must provide accurate, evidence-based, and timely feedback and support. Guidance on how users can ensure data syncing is optimized will help troubleshoot potential issues.	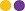
User Experience	Emotion	11.2. Reduce negative emotions	Prioritise user experience design as a core part of the DH development lifecycle to ensure the final product can compete with current market standards. Engage in iterative user experience testing prior to launch so that first impressions are good and lasting. Include simple gamification elements (such as streaks, points, coins), tied to incentive and reward structures and tracking of user goals, behaviors, and outcomes.	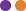

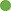
Community-based support and promotional activities.

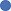
Brief counselling and provision of resources within primary care settings.

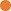
Variable incentives and rewards.

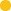
Policy and regulatory processes.

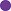
Prioritizing user experience.

#### Influencing uptake of DH solutions

Knowledge, social influences, and behavioral cuing are all MoA that underpin awareness of DH. Effective evidence-based BCTs that could therefore be used to increase uptake of DH include, for example, “providing information about health consequences” through community-based and interactive channels, delivering “prompts and cues” through mediums that have high user interaction and in areas with high footfall, and promoting “social support” through refer-a-friend incentives to increase the likelihood of trusted referrals through word-of-mouth. The weighing of benefits and burdens with respect to the adoption of DH is largely influenced by one’s knowledge, beliefs about consequences, and motivation. In turn, BCTs that may be effective include those related to natural consequences (e.g. information about health and emotional consequences, anticipated regret), comparison of behavior (e.g. pros and cons), and reward and threat (e.g. material incentives), which could be collectively addressed through opportunistic brief counselling, such as during routine health screening in, for example, a primary care setting. Beliefs about capabilities, skills, and environmental context and resources underpin accessibility. Here, providing “instructions on how to perform the behavior” and practical “social support” in a community setting, for example by providing digital literacy training as well as affordable access to DH solutions, through means tested or reimbursement schemes, may assist in broadening the adoption of DH in harder to reach communities. Finally, social influences and one’s attitude towards the behavior underpin trust in DH programs and apps, therefore ensuring that only “approved” solutions are promoted by a “credible source” (e.g. a trusted organization or person) could be achieved through enhanced policies and regulatory process to ensure only legally compliant and evidence-based solutions are made available.

#### Influencing sustained use of DH solutions

Behavioral cuing strategies, such as action planning, prompts, and habit formation, can effectively raise awareness of health behaviors, outcomes, and the benefits of using DH solutions, thereby promoting sustained use. Developers should integrate components that deliver these techniques into DH program and app design. Since the appraisal of benefits versus burdens is a continual process, it is essential to leverage knowledge, feedback processes, motivation, and reinforcement to ensure perceived benefits continue to outweigh the costs. Techniques like goal setting, self-monitoring, biofeedback, and feedback on behavior and outcomes align well with DH capabilities and should also be prioritized in their development. Furthermore, offering varied rewards and incentives that resonate with different core values may encourage continued engagement, since not all users respond to the same incentives. Implementing DH solutions and health monitoring tools within the healthcare system and linking them with electronic medical records will also allow healthcare professionals to enhance brief counselling and feedback processes leading to better integrated care and better management of health. One’s general attitude or belief about the trustworthiness of a DH solution influences its continued use. Ensuring that the data accurately reflects the individual and their situation is crucial, and efforts must be made to guarantee the credibility and accuracy of information and feedback. Finally, emotion is a mechanism of action that can underpin user experience, which is closely tied to engagement and, in turn, the potential recommendation of a DH solution to others. As such, ensuring high user satisfaction with DH programs and apps and “avoiding negative emotions” such as frustration, disappointment, or anxiety should translate to good long-term engagement and more word-of-mouth recommendations.

## Discussion

This study explored factors influencing DH adoption in a diverse population and identified strategies to support broader use for nationwide preventive healthcare. Our novel PPI approach allowed us, for the first time, to consider viewpoints from the people who are not using DH technologies, thereby giving us a unique perspective on the reasons for non-adoption and how they may be addressed. These findings are key to bridging the digital divide and realizing a “digital health for all” future.

### Uptake of DH solutions

A lack of awareness of available DH solutions remains a significant barrier to their uptake, even in Singapore, where digitalization is high. Our findings indicate a need for more extensive promotion and campaigns in the community to increase knowledge around available DH programs and apps and what benefits they can offer, especially using “offline” approaches among the older generations. Mass media campaigns can be effective in promoting health-related behaviors and our findings align with evidence that longer and more intense campaigns with message framing around social norms and targeting specific groups, may increase their effectiveness [27, 28]. Additionally, as shown elsewhere [29], we found social influences play a critical role in raising awareness and building trust in DH. Recommendations from family, friends, employers, or healthcare professionals can have a substantial impact on an individual's decision to adopt a DH solution. Leveraging this influence through strategies such as “refer-a-friend” initiatives, corporate social responsibility partnerships, or brief counselling within routine healthcare could be an effective means of promoting DH uptake. However, implementing such strategies would require coordinated, cross-disciplinary efforts to ensure that systems and partners are prepared to support these initiatives [30]. Moreover, the barriers experienced by healthcare providers in prescribing or promoting DH programs and apps to patients, such as workflow adjustments, inadequate reimbursement, and lack of training [31], must be addressed, and regulatory frameworks may be needed before DH solutions can be promoted in the healthcare setting.

Our findings also reinforce the importance of keeping barriers to uptake extremely low. Health behaviors are challenging for individuals to adopt and sustain, particularly when motivation is low [32]. Additional negative attitudes or ambivalence towards using DH technologies, despite their proven effectiveness in facilitating health behavior change and improving health outcomes [33], could further complicate uptake, especially within certain population groups. Individuals also constantly balance the perceived benefits of a DH solution against the effort or burden of using it. Research shows that perceived disease threat and health consciousness influence attitudes towards DH technologies and intentions to use them [29], at least initially. Thus, communicating the benefits of a DH solution at key decision-making moments and aligning with an individual’s perceived needs and benefits could ensure appraisal in favor of DH adoption. Monetary incentives and rewards can also reduce the perceived burden and promote uptake, as seen in studies from Singapore [23, 34] and elsewhere [35]. Nevertheless, unless policy shifts towards greater investment in preventive healthcare, financial reward schemes might not be sustainable in the long-term. Moreover, our findings indicate that monetary rewards may not appeal to everyone, therefore alternative reward structures that align with personal goals, values, and aspirations [36], such as charitable donations or tree planting, could increase the appeal and effectiveness of these incentives. Data-driven insights into user behavior and reward preferences could further optimize these systems in a cost-effective way.

Finally, our findings underscore the importance of addressing accessibility barriers, particularly concerning upfront costs, digital literacy, and language diversity, when developing new DH programs and apps or refining existing ones. Similar uptake barriers have been identified in a recent systematic review of DH use in culturally diverse populations [4]. Caution should also be paid to developing apps that rely on data syncing with wearable devices. Even when devices are offered free of charge (as in Singapore), access barriers remain, which limits their uptake. While the adoption of wearable devices will grow, they should not be a prerequisite for DH access.

### Sustained use of DH solutions

Sustaining adherence to DH programs and apps remains a significant challenge. One systematic review of mental health apps “in the wild” showed a rate of decline of more than 80% in the first ten days following app download and 30-day retention rates of only 3.3% [6]. There is evidence to explain the determinants of health app use and factors influencing adherence, such as personalization, integrity, utility, user-friendliness, technical stability, app design, and social and gamification features [5, 29, 37, 38]. However, our findings go one step further to explain why these factors are important and what strategies may be used to prevent disengagement.

We found that continued engagement with health apps hinges on factors such as user experience, app design, gamification, rewards, and accurate health monitoring. Decisions to adopt an app are strongly tied to satisfaction, as outlined in technology acceptance models [39, 40]. Users prefer simple, user-friendly apps, while overly complex ones lead to frustration and quick abandonment. Studies show that people form initial impressions of apps within four seconds, based mainly on design aesthetics and usability [41, 42]. Although, factors like perceived usefulness and compatibility may take longer to assess, they are also critical for sustained use. Aligning these aspects with individuals’ perceived needs and benefits, personalized health insights, and actionable feedback is crucial. Moreover, digital literacy is a moderating factor to consider [5, 43], and ensuring a person has the necessary skills to use the technology and understand the feedback it provides could be vital to their evaluation and adoption of an app.

The issue of sustained use extends beyond user experience alone. When the primary goal is interaction with the technology, principles of technology acceptance predominate, and usage is facilitated when tasks are made as effortless as possible—such as passive step tracking. However, when DH solutions serve to promote “offline” health behaviors, different factors come into play. Here the relationship between DH use and behavior change is not necessarily causal and can be moderated by individual [29] and time-varying factors [44]. For instance, participants in our study reported discontinuing app use when they felt they no longer “needed” the app or when they did not “see the benefit.” While such disengagement is concerning for content delivery, it does not necessarily indicate failure, especially if the app has already fulfilled its purpose in facilitating behavior change.

Another consideration is that an individual's motivation and health needs fluctuate over time [45]. Participants in this study highlighted that health tracking, reminders, gamification, and rewards together form an effective strategy for sustained engagement with DH solutions, aligning with findings from systematic reviews [5, 46] and engagement theories such as the “Hooked Model” [47]. Leveraging longitudinal health tracking data collected through apps and wearables can offer insights into behavioral patterns [48], enabling more precise, personalized, and predictive interventions [49], especially when adopting machine learning and large-language model approaches. Using these data to send just-in-time adaptive interventions—delivered at moments when users are most receptive and in need [50–52] - may further enhance engagement, especially when connected to personally relevant goal-directed activities in everyday life [53]. Gamification strategies, such as streaks and challenges, have been shown to enhance motivation and encourage continued use, making DH programs and apps more effective [54]. Financial rewards appear to be highly effective in stimulating initial uptake if they are ‘worth the effort’ to the user, but their effectiveness in sustaining engagement is still debated [23]. While there is some evidence that people do respond to this strategy [35], more research is needed to better understand the effects of differentiated incentive and reward schemes on long-term DH engagement [55].

Finally, financial considerations, such as the cost of wearable devices and ongoing subscription fees, also emerged as significant barriers to sustained use, highlighting the importance of affordability in ensuring equitable access to and use of DH. Despite the widespread use of freemium business models in the health and wellness industry [56], participants in this study were dissatisfied with apps that adopted the approach, citing it as the reason for their disengagement. Other research has also highlighted the ethical issues caused by withholding health-related support to those in need—particularly concerning mental health—based on financial payment [56]. Such practices may contribute to health disparities. The introduction of certification, quality assurance, and approvals of DH solutions from regulatory bodies leading to reimbursement through the healthcare system may make such paid-for services more accessible.

### Strengths and limitations

Strengths of this study include the participatory research approach which involved laypeople with relevant language skills, allowing us to capture the perspectives of a diverse group, including individuals who typically do not participate in health research. These varied viewpoints, especially from those who are not using DH, offer valuable insights into the reasons for non-adoption and potential strategies to address them. It should be noted that, when contrasting our sample characteristics with Census data, we may have over-sampled younger people (aged 21–40 years) and under-sampled those without formal secondary education or with higher household incomes (>$10 000 SGD). Furthermore, the findings should be considered alongside the highly digitized landscape in Singapore which may limit generalzability to other countries. Nonetheless, the study provides important lessons for public health and industry professionals on the key factors influencing DH adoption.

### Recommendations

Through the analysis of the behavioral MoA, we identified five key strategies that could be used to facilitate DH adoption by diverse and multicultural populations. These are: (1) *community-based support and promotional activities*: targeted and regular mass media campaigns, point-of-choice promotion materials in areas with high footfall, cross-promotional strategies via other public service apps, and strategies to encourage referral from trusted sources (such refer-a-friend incentives); (2) *brief counselling and resources within healthcare settings*: provision of information, resources, and motivational support that can assist people to appraise the benefits of using a DH solution and how it can be used to manage health. Support should be personalized where possible, based on biofeedback, electronic medical records, and health monitoring data, and embedded within the healthcare system to ensure integrated care; (3) *variable incentives and rewards*: provision of incentives and rewards that appeal to different core values and can be sustained long term and are easy to redeem; (4) *policy and regulatory processes*: introducing regulatory processes whereby only DH solutions that are approved and legally compliant (by relevant authorities and standards) can be made available to consumers; and (5) *prioritizing user experience:* following principles of user centred design and prioritizing the inclusion of digital components that facilitate self-regulation of health and behaviors as well as gamified elements that link to rewards.

## Conclusion

This study provides valuable insights into the complex interplay of factors influencing the adoption of DH. By addressing barriers related to awareness, affordability, accessibility, and user experience, stakeholders can enhance the effectiveness and reach of DH solutions, ultimately promoting population-wide health and well-being. Further research and targeted interventions are warranted to address individuals’ diverse needs and preferences across different demographic groups and to better understand the relationships between DH engagement and health behavior change and how these vary over time. Finally, it is essential that the principles of user-centred design are prioritized at an early stage of any DH development, and smooth onboarding processes and overall user experience should meet, or potentially *exceed,* the expectations of the target user group to maximize engagement.

## Supplementary Material

ibaf010_suppl_Supplementary_Files_1

ibaf010_suppl_Supplementary_Files_2

ibaf010_suppl_Supplementary_Files_3

## Welfare of Animals

This article does not contain any studies with animals performed by any of the authors.

## Transparency Statements

*Study Registration:* This study was not formally registered*. Analytic Plan Pre-Registration:* The analysis plan was not formally pre-registered. De-identified data from this study are not available in a public archive. De-identified data from this study will be made available (as allowable according to institutional IRB standards) by emailing the corresponding author. *Analytic Code Availability:* There is no analytic code associated with this study. *Materials Availability:* Some of the materials used to conduct the study are presented in a public archive: https://osf.io/6dfeu/
