# Supplementary material for: Understanding and overcoming barriers to digital health adoption: a patient and public involvement study
Source: Transl Behav Med. 2025 Apr 1;15(1):ibaf010. doi: 10.1093/tbm/ibaf010 (PMC11959363; doi:10.1093/tbm/ibaf010)
Supplement: ibaf010_suppl_Supplementary_Files_2 [file ibaf010_suppl_supplementary_files_2.docx]

Digital Health for Health Promotion: PPI Hawker Sessions

1. Opening line & permission to speak
2. Brief study details & assurance of anonymity
3. Digital health overview:
   1. Do you use any health app/digital health devices/participate in any health programmes online?
   2. What is “digital health” to you?
4. Distribute participant chit. Instruct participants to indicate any programmes or apps used
5. Discussion:

| **Have used apps** | | | **Have not used apps** | |
| --- | --- | --- | --- | --- |
| Currently still using | | Stopped Using | Never heard/Don’t know | Heard of, but do not use |
| 1. What made you download and use the app? 2. When did you start using this app? 3. How long have you been using/did you use the app for? 4. Have you noticed any changes since you’ve used this app? 5. Any difference to your life? | | | 1. Do you maintain routines or habit to stay healthy? 2. Do you keep track of your healthy habits? 3. How do you tell if your routine stays constant/adequate e.g. sufficient pacing/distance for running or walking to maintain high heart rate 4. Is there anything you wish you could be doing more to stay healthy? | |
| - How often do you use this app? - What do you like about it? - What makes it useful? - Any particular feature or function? - Do you think you will continue to use this app for a long time? - Why/Why not? | | - Why did you stop using this app? - What would motivate you to go back to it? - If there is something you can change about it, what would it be? - Is there another app that appealed to you more? If so, which one and why? | - What does digital health mean to you? - Is there a reason why you have not engaged in health apps/programmes? - What would make it easier/more appealing for you to use it? - Do you use other apps for your health and wellbeing? - What would an ideal support for health promotion look like to you? | - How did you hear about this app/programme? - Do you know anyone who uses this? - Why do you not use it? - Which feature of the app/programme makes it ___ for you? - What would make you want to use it? |
|  | | | | |
| Other questions: | - (*If discussion is centred on physical activities only*) How about other healthy habits e.g. monitoring diet, meditating? - If there were a health app that you could create for yourself, what would that look like? - What are some programmes and applications you hope to see be developed? - Anything else you would like to add or comment on? Any questions? | | | |
