# Supplementary material for: Understanding and overcoming barriers to digital health adoption: a patient and public involvement study
Source: Transl Behav Med. 2025 Apr 1;15(1):ibaf010. doi: 10.1093/tbm/ibaf010 (PMC11959363; doi:10.1093/tbm/ibaf010)

| HPB Programmes | | | |
| --- | --- | --- | --- |
| Healthy365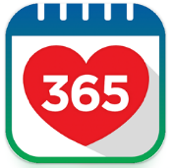 | 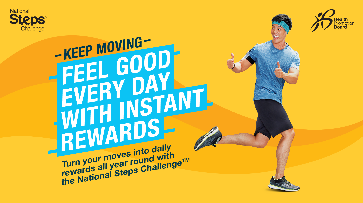 | 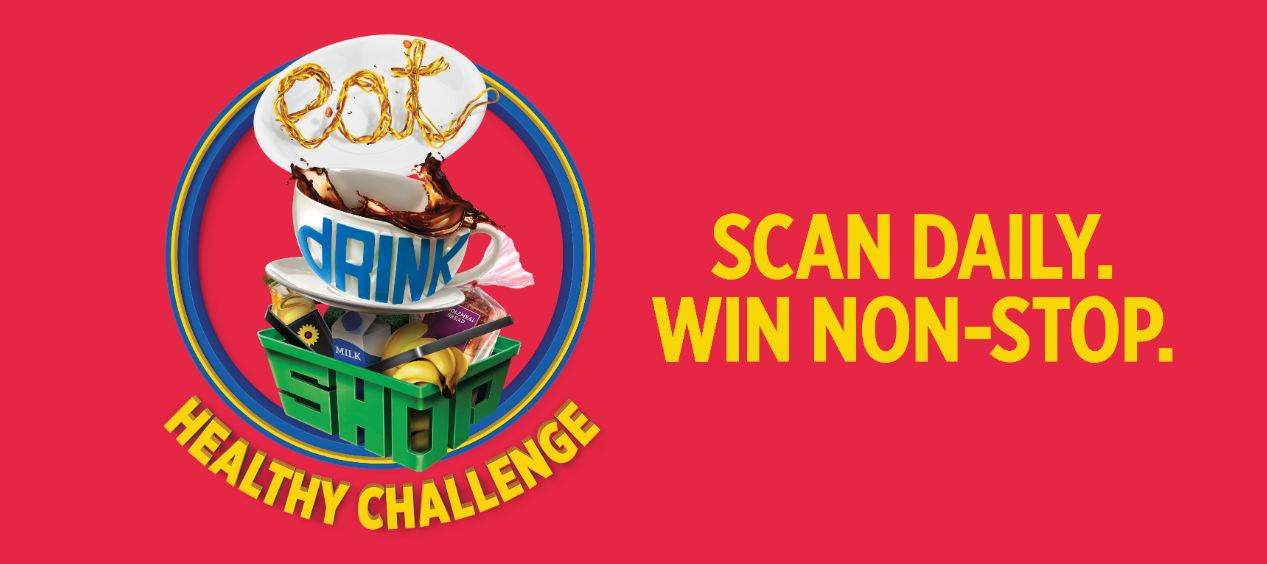 | 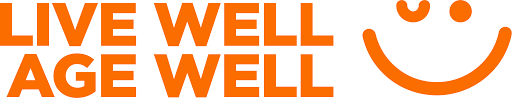 |
| 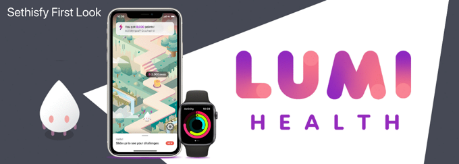 | | 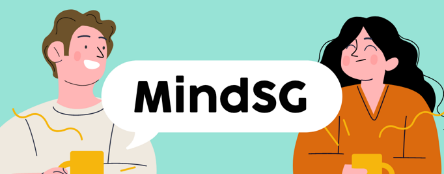 | 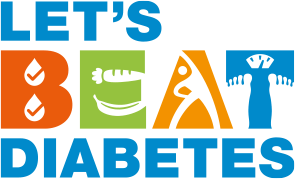 |

| Commercially Available | | | | | | |
| --- | --- | --- | --- | --- | --- | --- |
| Step Tracker  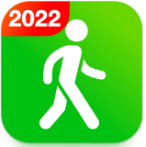 | SweatCoin  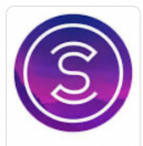 | Fitbit  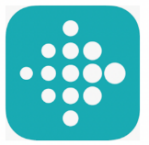 | MyFitnessPal  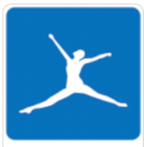 | Lifesum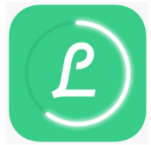 | Noom  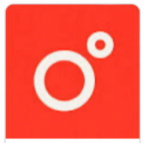 | Calorie Counter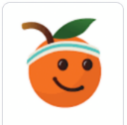 |
| Apple Fitness/Samsung Health/Google Fit  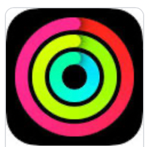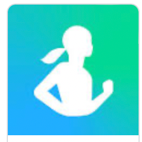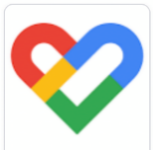 | | | Wysa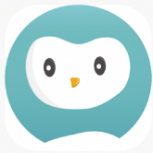 | Calm  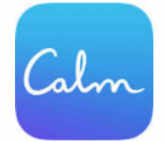 | Woebot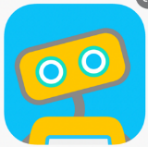 | Headspace  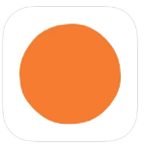 |


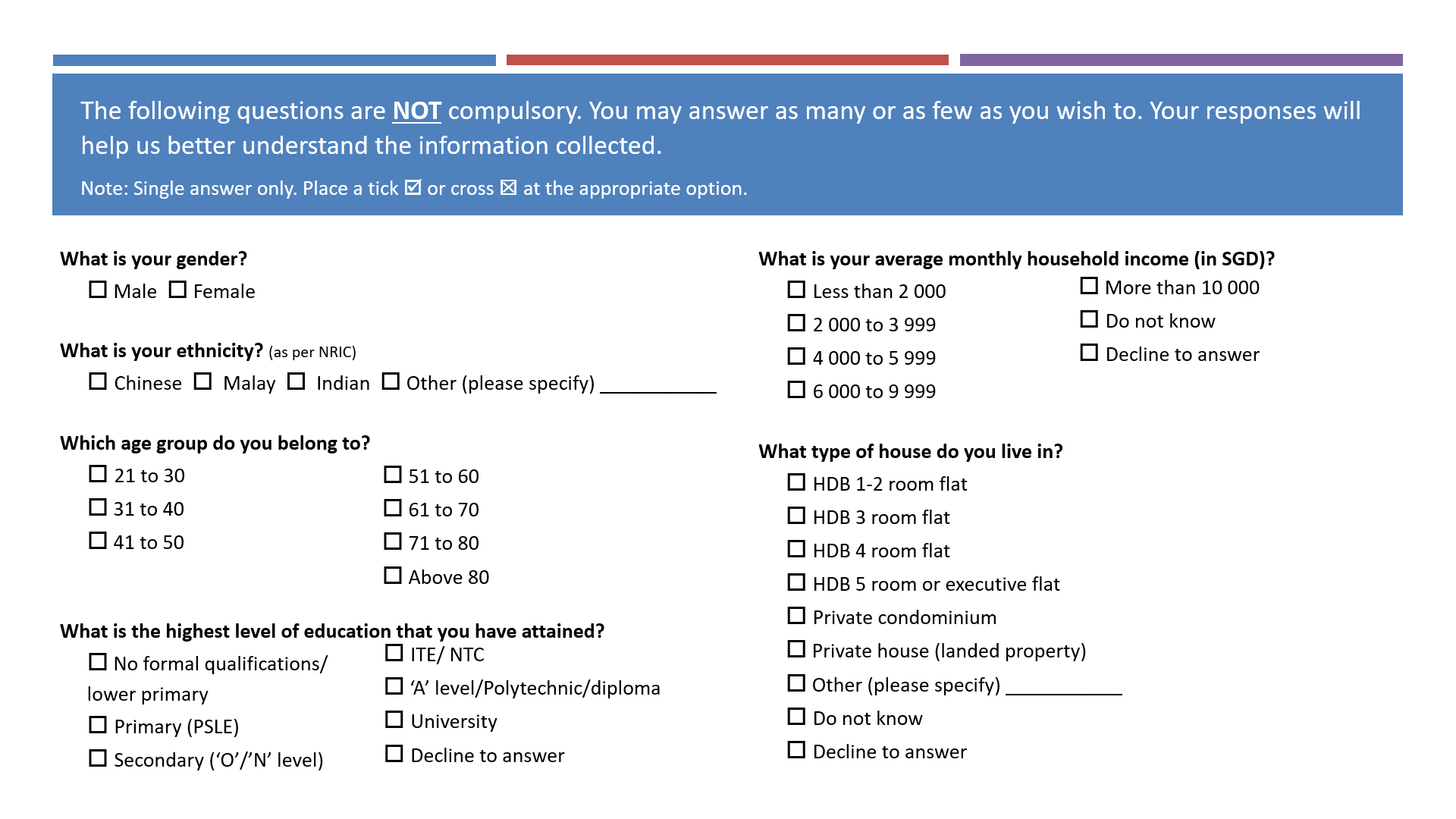


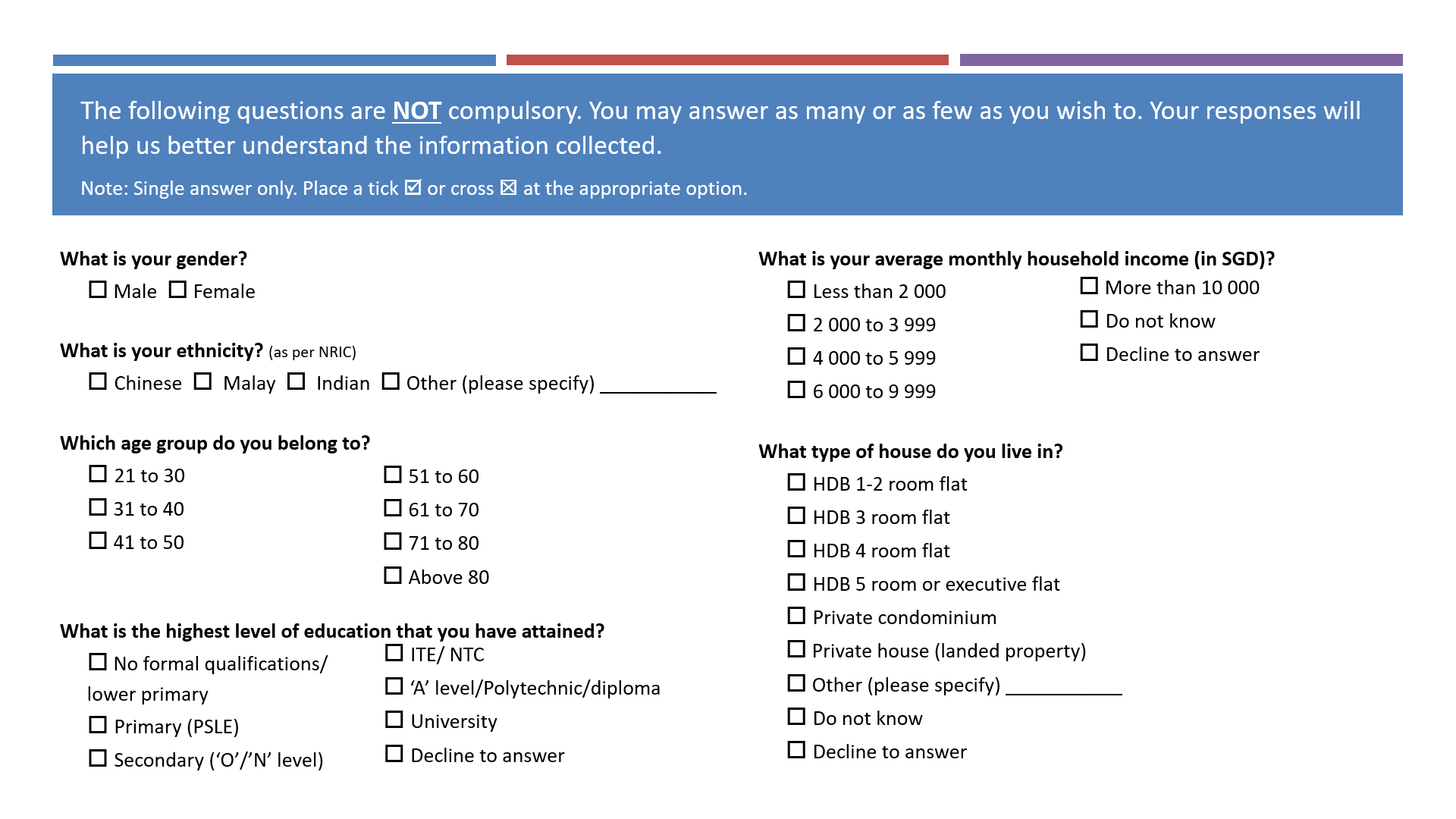

Supplement: ibaf010_suppl_Supplementary_Files_3 [file ibaf010_suppl_supplementary_files_3.docx]
